# Reply to Rizzo et al.

**DOI:** 10.1055/a-2867-2365

**Published:** 2026-06-02

**Authors:** Kambiz Kadkhodayan, Shayan Irani

**Affiliations:** 1 Center for Interventional Endoscopy 440172 AdventHealth OrlandoOrlandoFloridaUnited States; 2 Gastroenterology and Hepatology 7289 Virginia Mason Medical CenterSeattleWashingtonUnited States



Dear Editor,

We sincerely thank Rizzo et al. for their thoughtful and constructive comments regarding our recently published pilot feasibility study. We appreciate their careful reading of the manuscript and their recognition that this work provides an important proof-of-concept for creating a durable, stent-independent endoscopic anastomosis.

In retrospect, the manuscript wording may have implied that completion of Step 2 was universally intended in all patients. In practice, this second stage was conditional upon interval clinical assessment. In the two patients with malignant progression, the decision not to proceed with Step 2 was intentional and clinically driven, rather than due to technical inability. Because no attempt was made at a second step, these cases were not adjudicated as technical failures. Accordingly, the technical success rate was 100% for dual-stent placement (6/6) and 100% for patients in whom the second step was undertaken (4/4).

With respect to heterogeneity of the cohort, we agree that inclusion of both benign and malignant etiologies, as well as variability in baseline anatomy, timing of the second lumen apposing metal stent (LAMS) placement, and stent fixation technique limit extrapolation of the results. This heterogeneity, however, reflects the pilot nature of the study. Our primary objective was not to establish comparative efficacy in a homogenous cohort of patients, but rather, to determine whether the dual-LAMS construct could be created safely across real-world scenarios and whether a durable gastrojejunal conduit could persist after stent removal. We agree with the authors that procedure standardization would improve reproducibility in further studies.

Importantly, this study demonstrates that a dual-LAMS construct can mature into a stent-independent gastrojejunal anastomosis. As highlighted by Rizzo et al, this approach may be particularly relevant in benign disease where a prolonged indwelling stent is undesirable. These findings remain preliminary, however, and the procedure should be undertaken with caution, in carefully selected patients, at centers with advanced endoscopic expertise and after a multidisciplinary evaluation given the technical complexity and potential for adverse events. We agree that further studies with long-term follow-up are warranted to study safety, durability of the anastomosis, clinical applicability, and efficacy of this approach, ideally within experienced centers with expertise in EUS-guided gastroenterostomy.

We thank the authors again for their kind and insightful comments, and for contributing to the ongoing scientific discussion in this area.

Sincerely,

Kambiz Kadkhodayan, MD

Shayan Irani, MD

**Publication note**

Letters to the editor do not necessarily represent the opinion of the editor or publisher. The editor and publisher reserve the right to not publish letters to the editor, or to publish them abbreviated or in extracts.

